# *In-House* Innovative “Diamond Shaped” 3D Printed Microfluidic Devices for Lysozyme-Loaded Liposomes

**DOI:** 10.3390/pharmaceutics14112484

**Published:** 2022-11-16

**Authors:** Federica Sommonte, Edward Weaver, Essyrose Mathew, Nunzio Denora, Dimitrios A. Lamprou

**Affiliations:** 1 Department of Pharmacy-Pharmaceutical Sciences, University of Bari Aldo Moro, Orabona Street, 4, 70125 Bari, Italy; 2 School of Pharmacy, Queen’s University Belfast, Belfast BT9 7BL, UK

**Keywords:** additive manufacturing, digital light processing, microfluidics, 3D printing, chips, liposomes, enzymes

## Abstract

Nanotechnology applications have emerged as one of the most actively researched areas in recent years. As a result, substantial study into nanoparticulate lipidic systems and liposomes (LPs) has been conducted. Regardless of the advantages, various challenges involving traditional manufacturing processes have hampered their expansion. Here, the combination of microfluidic technology (MF) and 3D printing (3DP) digital light processing (DLP) was fruitfully investigated in the creation of novel, previously unexplored “diamond shaped” devices suitable for the production of LPs carrying lysozyme as model drug. Computer-aided design (CAD) software was used designing several MF devices with significantly multiple and diverse geometries. These were printed using a high-performance DLP 3DP, resulting in extremely high-resolution chips that were tested to optimize the experimental condition of MF-based LPs. Monodisperse narrow-sized lysozyme-loaded PEGylated LPs were produced using in-house devices. The developed formulations succumbed to stability tests to determine their consistency, and then an encapsulation efficacy (EE) study was performed, yielding good findings. The in vitro release study indicated that lysozyme-loaded LPs could release up to 93% of the encapsulated cargo within 72 h. Therefore, the proficiency of the association between MF and 3DP was demonstrated, revealing a potential growing synergy.

## 1. Introduction

Nanomedicine has become one of the most highly explored investigation fields in recent years. It involves the application of nanotechnology in the medical area, and its great potential arises from the fact that it covers various areas of research, including biomedicine, biomaterials, and pharmaceutical sciences to solve deficiencies related to classical medicine [1,2,3]. Thus, there has been a widespread investigation of nanosystems as emerging drug delivery systems (DDSs) according to their potential compared to classical drug delivery methods [4]. The enormous progress in DDS research is clearing the way for a new concept of therapeutic treatment, which is based on the use of nanovectors that may increase the activity of the drug itself and reduce aspects such as: systemic side effects, the dose to be administered, and, indirectly, the cost compared to classical therapeutic schemes [5,6].

Lipid nanoparticles (LNPs), and among them liposomes (LPs), represent one of the most explored DDS. Recently, there has been a growing exploration of the use of LNPs in therapy, especially in the areas of cancer treatment and vaccinology [7,8]. The emergency due to the COVID-19 pandemic prompted the scientific community to exploit these new formulations quickly and efficiently, leading to the very rapid development and commercialization of mRNA-based vaccines using LNPs as selected vectors [9]. This ground-breaking phenomenon has highlighted their potential applications in therapy. LPs are essentially composed of phospholipids that self-assemble to form vesicular structures in which the lipid bilayer surrounds an aqueous core [10]. Moreover, in addition to the application of one or more phospholipids, they involve the use of cholesterol to support the architecture of the lipid chains while modulating their rigidity/flexibility and the release of the encapsulated drug [10]. The application of physiological lipids improves their biocompatibility, well tolerability, and non-toxicity [11]. The physicochemical properties of LPs can be modified to allow them to specifically target the site of interest, either through passive targeting by the enhanced permeability and retention (EPR) effect or actively by adding specific ligands that recognize a molecular pattern to the targeted tissue [12]. In addition, it is possible to make them long lasting in the circulation by the process of PEGylation or by the use of (poly)-ethylene glycol (PEG)-conjugated phospholipids that can avoid rapid clearance by the reticuloendothelial system (RES) [10]. Finally, their versatility in encapsulating both hydrophilic and hydrophobic drugs has led to many diverse applications [10,13].

Despite the advantages, several related problems have made their development still challenging. Among them, the classical synthesis of LPs is unreliable; thus, it has become essential to find innovative emerging methods that enable safe, fast, and high-level batch-to-batch reliability production. To prevail over these challenges, applications of microfluidic (MF) technique to the development of new formulations in NM have successfully been exploited [14,15,16]. MFs have emerged in recent years as a suitable tool for biosensing and DDSs’ production, since MF removes some of the critical issues that prevent NM from becoming a concrete reality [17]. The *in-flow* technique is scalable and allows for a drastic reduction in production time, greatly improving the quality of the finished product [2]. As MF ensures strict control over all operational parameters, the result can be optimized with experimental trials, and once optimal conditions are identified, batch-to-batch variability is eliminated [18,19]. Furthermore, in comparison to conventional methods, MF-based processes ensure less consumption of solvents that currently constitute the 80–90% of all materials used during pharmaceutical production. This technique can minimize the environmental impact, leading the way to a more sustainable approach [20].

Nevertheless, even this technique has certain limitations that are closely related to the devices used. Commercially available MF devices are extremely expensive and do not easily allow for flexibility in design and materials, which largely limits their use in research [21]. Therefore, in parallel with the development of MF, there has been a growing demand for manufacturing processes that enable different devices to be developed that are cheaper, smarter to produce, and have a customizable geometry, hence the emerging development of three-dimensional printing (3DP) technology associated with MF.

Three-dimensional printing is a technique suitable for massive commercial and technological applications. In the last decade, additive manufacturing (AM), which encompasses a layer-by-layer (LbL) process to manufacture solid artifacts, has become particularly promising around personalised medicine and pharmaceutical technology [22]. Specifically, there is a move towards ever-closer automation in the production of affordable and customisable 3DP solid pharmaceutical objects. In parallel, 3DP is also a valuable support in the production of MF devices applicable to the synthesis of innovative formulations, lab-on-a-chip devices, and organo-mimetics [23,24,25].

Soft lithography, in which a polymer such as polydimethylsiloxane (PDMS) is cast onto rigid substrates, has been widely used in the past to fabricate MF devices. This process requires subsequent fixing and alignment of various components, increasing the probability that dust or operator error could have a negative impact on the output. For this reason, other techniques have emerged as stronger, which in one fast, reproducible, and economical process ensures high quality of the final product [26]. Thus, since 2010, resin 3DP techniques associated with high-performance MF devices have been highlighted, involving the use of UV–visible light to induce LbL polymerisation reactions of photosensitive resins [26,27].

Lately, digital light processing (DLP) printers have been extensively developed, which exploit UV light and consent to layer polymerization along the light path. Although there are different DLP printers, all share the same working system. These are composed of a photocurable precursor, a printing platform, and a light source (photoinitiator). The significant difference between stereolithography (SLA) and DLP printers is that in the former case, the light source is a point laser, whereas DLP employs a digital projector. The photopolymerization of the resin occurs due to the photons emitted by the photoinitiator [23,28]. The intensity of the light beam is planar at the interface and is dynamically adjusted by the software associated with the printer. Printing takes place LbL, but each layer is polymerized simultaneously by the photoinitiator, making the production faster. In addition, DLP does not require high temperatures and ensures extreme precision compared to other commercially available printers [29]. This technical improvement has provided a winning choice for the selection of this type of methodology in various fields of application, including audiology, dentistry, and application to the production of MF devices [23,26]. Hence, the capability to print low-cost high-resolution microchannels with intricate geometries, while being able to customise the design according to requirements using computer aided design (CAD) software, has been revealed to be innovative and successful to be applied to MF [27].

In the field of pharmaceutical nanotechnology, this collaboration overcomes the obstacles that complicate short-term results. The great advantage of the two techniques’ combination lies in the fact that with DLP 3DP, it is possible to have ready-to-use devices, made of inert and resistant material, reusable and completely customised to the individual requirements. By combining 3DP and MFs, it is possible to reach innovative formulations with the desired characteristics based on the geometry of the self-produced device, in a reproducible, and above all fast and economical manner [30]. This consents to carrying out comparative studies between the different device’s geometries to customise one’s own according to the intended result. This is feasible without having to adapt to commercially available devices, which generally require long waiting times for delivery and are very expensive, as they cannot guarantee such a wide variety of choices between designs as the possibility of a self-created one.

The originality of this study is found in the tangible illustration of the potent synergy between 3DP and MF applied to experimental nanomedicine. This approach is an intriguing novelty considering several key points: first of all, new structured micromixing geometries have been fully customized and generated using CAD software; as a second aspect, these devices were printed using a high-performance DLP 3DP, which is still underutilized for MFs application, resulting in one-step extremely high-resolution printed ones, which were tested producing empty LPs to optimize the total flow rate (TFR) and fluid rate ratio (FRR) parameters. Finally, the 3D-printed devices were successfully applied to the generation of LPs carrying lysozyme as a model enzyme, overcoming the limitations that prevent biomolecule-loaded DDS to be produced.

Formulations related to most performing devices were subjected to long-term stability studies, and subsequently, encapsulation efficacy and release studies were carried out to assess the validity of MF-3DP synergic protocol. To the best of our knowledge, there are still few examples in the literature where fully customized *in-house* 3D-printed devices have been successfully applied to the production of innovative formulations carrying a biomolecule, implementing a novel protocol suitable for future applications.

## 2. Materials and Methods

### 2.1. Materials

**Figure 1:**
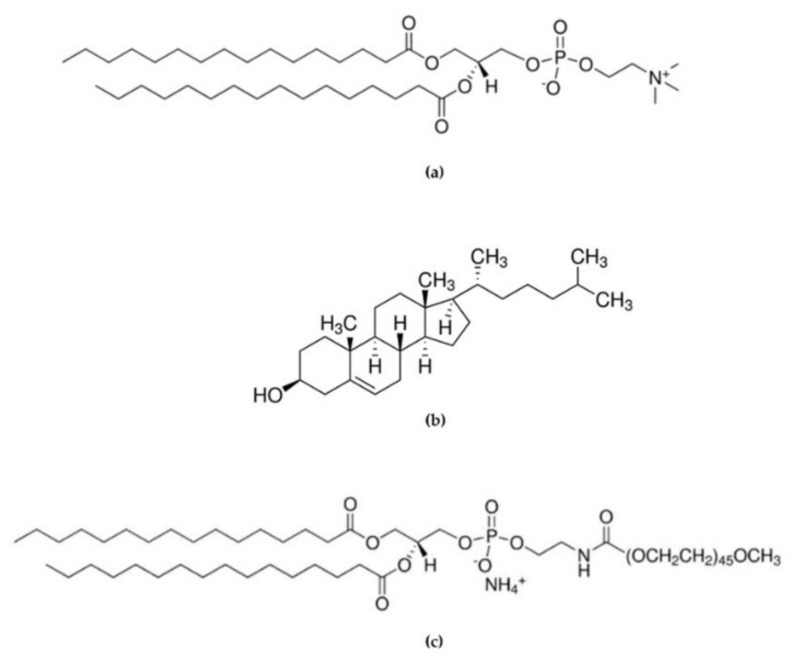
Chemical structure of (**a**) 1,2-Dipalmitoyl-sn-glycero-3-phosphocoline (DPPC), (**b**) Cholesterol (Chol), and (**c**) 1,2-dipalmitoyl-sn-glycero-3-phosphoethanolamine-N-[methoxy(polyethyleneglycol)-2000] (16:0 PEG2000 PE).

All chemicals were the highest purity available and were used as received without further purification or distillation. 1,2-Dipalmitoyl-sn-glycero-3-phosphocoline (DPPC, >98%), Cholesterol (>99%, stabilized with α-Tocopherol) were purchased from Tokyo Chemical Industry UK Ltd (Belgium). 16:0 PEG2000 PE 1,2-dipalmitoyl-sn-glycero-3-phosphoethanolamine-N-[methoxy(polyethyleneglycol)-2000] (ammonium salt) was purchased from AVANTI^®^ Polar Lipids, INC (Molecular structures can be seen in Figure 1). Lysozyme (from chicken egg white, lyophilized powder, protein ≥90%, ≥40,000 units/mg protein), tablets of phosphate-buffered saline (PBS, pH 7.4), ethanol, isopropyl alcohol (IPA), and acetonitrile ≥99.8% were obtained by Sigma-Aldrich (Steinheim, Germany). Microfluidic devices were fabricated using an Asiga MAX UV 3DP and PlasCLEAR resin (Asiga, Alexandria, Australia). 

### 2.2. Design and Manufacturing of 3DP Chips

Microfluidic devices’ (chips) designs were created using an open-source computer-aided design (CAD) software (TinkerCAD, USA). The created devices were converted to .STL files and uploaded to the slicing software Asiga Composer (Version 1.2). The designs were placed at an angle of 0°, and no print supporters were added.

The printer used was the Asiga Max UV (Asiga, Alexandria, Australia). The devices were printed at a resolution of 0.0025 mm, using PlasCLEAR resin (Asiga, Alexandria, Australia).

At the end of the printing phase, the devices were removed from the building plate and bathed into isopropyl alcohol (IPA) to remove excess resin inside the channels. Subsequently, the devices were sonicated for 8 min using Ultrawave QS12 Ultrasonic Bath (Dorset, UK). At the end of the process, the devices were left to air dry, and once the solvent had evaporated, they were cured for 20 min in a 385 nm UV chamber (Asiga Flash, Melbourne, Italy) as recommended by the printer guidelines. Optical microscope images were obtained using a Leica EZ4W with software Leica.

### 2.3. Production of LPs by MFs

**Figure 2:**
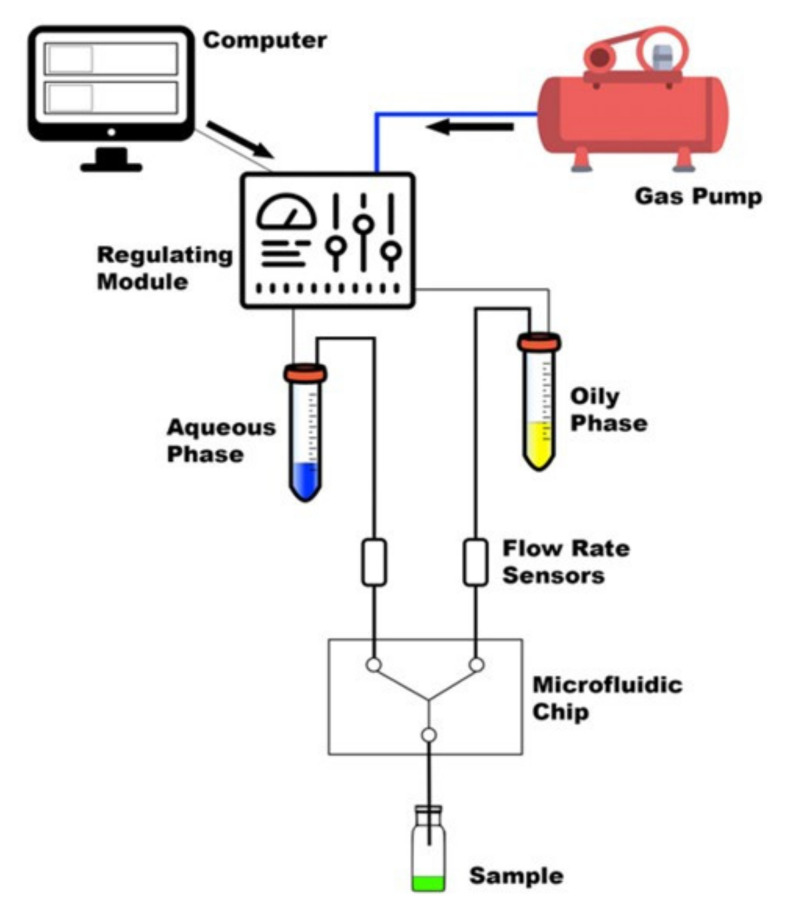
Summary of the main components of MF system [34].

LPs were produced using a dolomite microfluidic system, and the TFR and FRRs were monitored using two Mitos Flow Rate Sensors (0.2–5 mL min^−1^) [31]. A simple schematic of the MF setup can be seen in Figure 2. Phospholipids (DPPC, 16:0 PEG2000 PE) together with cholesterol were dissolved in ethanol (>99%) in a ratio of 2:1, respectively, at a final concentration of 1 mg mL^−1^. This ratio and concentration of lipids can achieve nanosized and consistent LPs, as investigated in a previous study [32,33].

The lipid solution was sonicated to facilitate its complete dissolution. The aqueous phase is phosphate-buffered saline (PBS, pH = 7.4). The two phases were injected into the several *in-house* produced microfluidic devices testing different FRRs and TFRs. 

The production of lysozyme-loaded LPs (L-LPs) was carried out by solubilizing lysozyme in the aqueous phase in a range concentration of 0.3–0.7 mg mL^−1^. Optimal production conditions were proven (data not reported here) until the most performing FRR (5:1) and TFR (6 mL min^−1^) were defined. The schematic set up of MF system is shown in Figure 2.

### 2.4. Physicochemical Characterization of LPs

Dynamic light scattering (DLS) was employed to assess the size and polydispersity index (PDI) of LPs, using a Nanobrook Omni particle sizer (Brookhaven Instruments, Holtsville, NY, USA). Each measurement was run in triplicate by performing a 1:95 dilution in PBS (pH 7.4). The ζ-potential was assessed using the same instrument through phase analysis light scattering (PALS) studies.

### 2.5. Stability Studies

Stability studies were performed at fixed time intervals (e.g., 0, 7, 14, 21, and 28 days) for up to four weeks. Stability was assessed at 4 °C to mimic the formulation’s storage condition and at 37 °C to mimic physiological conditions after administration. At predetermined time intervals, the size, PDI, and ζ-potential of each formulation were analysed. All measurements were performed in triplicate and mean, and standard deviation was calculated from the obtained data.

### 2.6. Encapsulation Efficacy and In Vitro Release Study

To determine encapsulation efficacy (EE), each experiment was carried out in triplicate. Briefly, 1 mL of lysozyme-loaded LPs was centrifuged at 14,800 rpm for 30 min. After centrifugation, 1 mL of supernatant was removed and replaced with 1 mL of PBS (pH 7.4). For the second time, samples were centrifuged under the same condition used previously. The resulting supernatants were collected and used for the calculation of EE following Equation (1).

(1) $$EE\;\%=\frac{\mathrm{Total}\;\mathrm{Amount}\;\mathrm{Of}\;\mathrm{Enzyme}\;\mathrm{Added}\;\left(\mathrm{mg}\right)-\mathrm{Amount}\;\mathrm{Of}\;\mathrm{Unencapsulated}\;\mathrm{Enzyme}\;\left(\mathrm{mg}\right)}{\mathrm{Total}\;\mathrm{Amount}\;\mathrm{Of}\;\mathrm{Enzyme}\;\mathrm{Added}\;\left(\mathrm{mg}\right)\;}\;\times \;100$$

The obtained sediment was resuspended in 1 mL of PBS, and it was used for enzyme release studies. This was performed utilizing dynamic dialysis (SpectraPOR^®^, 50kDa, Fisher Scientific, Milano, Italy). Briefly, 1 mL of each sample was placed within the dialysis tube, closed at both ends and placed in a PBS bath (7 mL, 37 °C). Release samples were collected as 0.5 mL aliquots at fixed time points (e.g., 30 min, 1, 2, 3, 4, 24, 48, and 72 h) and replaced with the same amounts of PBS kept at 37 °C. Studies of EE and release from LPs were performed utilizing ultraviolet high-performance liquid chromatography (UV-HPLC). For UV-HPLC, the Agilent Technologies 1220 Infinity LC system (CA, USA) was used to assess the amount of lysozyme. A C18 ODS HYPERSIL column (250 × 4.6 mm, particle size 5 μm) from Thermo Scientific (USA) was used. The method was modified from a study in the literature [35]. The mobile phase of the gradient system consisted of water/trifluoroacetic acid 0.1% (*v*/*v*) (A) and acetonitrile/trifluoroacetic acid 0.1% (*v*/*v*) (B). A flow rate of 1 mL/min was applied, using the following gradient profile: 100% A for 20 min up to 50% B within 2 min, where the solvent was kept constant for 3 min, and then again up to 100% B within 2 min where the solvent was kept constant for 12 min. Column temperature: 45 °C. Mobile phase flow: 1 mL min^−1^. Each sample was run for thirty-five minutes, and it was injected with 50 μL. Absorbance was recorded at λ = 280 nm [35]. All measurements were performed in triplicate, and mean and standard deviation were calculated by the obtained data.

### 2.7. Statistical Analysis

Data are presented when needed as a mean value, displaying a ± standard deviation (SD) value. All data were obtained in triple replicates unless stated otherwise. When comparing data sets for statistical significance, one-way ANOVA analysis was performed (Version GraphPad Prism 8.0.2), adhering to a p value of ≤0.05. Statistical differences are reported as follow: ns = *p* value > 0.05; * = *p* < 0.0332; ** = *p* value < 0.0021; *** = *p* value < 0.0002; **** = *p* value < 0.0001.

## 3. Results and Discussion

### 3.1. Rationale behind the Design of MF Devices

In this work, the 3DP MF devices were purposely engineered to be applied to LP production. By its definition, MF is “The science and technology of systems that process or manipulate small amounts of fluids (10^−9^ to 10^−18^ L) using channels ranging in size from tens to hundreds of micrometres” [36]. Microscale systems initially found practice in chemical analysis, and then, their beneficial potential in the field of technological application was investigated [36]. By miniaturizing devices, fluids are forced to flow into microchannels, and this leads to significant differences compared to macroscopic systems. In the latter case, the main force is turbulence, while in microchannels, fluid layers tend to stream in parallel, generating the laminar flow state (Reynolds number < 10^2^), and therefore, chemical reactions depend on diffusion processes across the interfaces of fluids and not convective processes [37]. Thus, in a laminar flow condition, there is strict control over the interdiffusion and the uniform mass transfer results in nanosized monodisperse vesicular formulation [38,39].

Based on the aim of producing high-quality monodisperse LPs, guidelines for the design of MF devices were applied. As reported in the literature, it is recommended that the produced chips should be similar in size to typical microscope stages (i.e., width = 25–50 mm and length = 75 mm). Inlets microchannels should be designed long enough (i.e., 5–10 mm) to ensure the formation of a predictable laminar flow before the meeting of the two phases [40].

**Figure 3:**
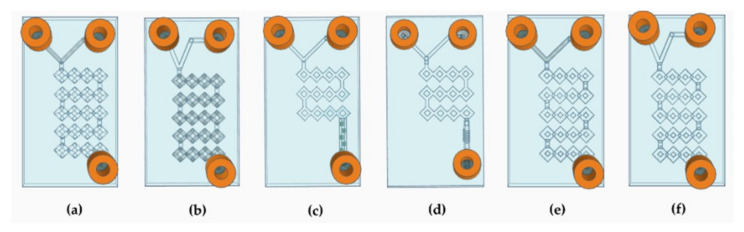
Schematic representation of the devices’ CAD design. (**a**) Representation of device 1. (**b**) Representation of device 2. (**c**) Representation of device 3. (**d**) Representation of device 4. (**e**) Representation of device 5. (**f**) Representation of device 6.

Following the above-mentioned evidence [40], it was decided to project various devices (Figure 3) that had all the required properties.

Since no external forces of flow perturbation were applied, these were passive MF devices in which the achievement of an adequate degree of mixing was exclusively due to the intricate internal architecture. In our case, parallel lamination micromixers (Y- and T-shaped), in which fluids are injected into separate inlets and then reunite in a long main channel, were designed. This type of chip is structured with channels long enough to generate adequate mixing while keeping low Reynolds number [41]. In some cases, laminar flow is not sufficient to create an adequate degree of mixing. This problem may be solved by increasing the FRR and TFR, but this approach results in greater solvent consumption. Therefore, to achieve quicker mixing under laminar flow conditions, it is possible to exploit the phenomenon of chaotic advection [37]. Chaotic advection can be generated by modifying device’s geometry, using tortuous channels, or by adding obstacles, bas-reliefs, or grooves. This approach causes the flow to be constantly stretched and reoriented, greatly increasing the mixing between phases even though the flow remains laminar (Reynolds number < 10^2^) and there are no dispersive phenomena [37].

Therefore, to increase the efficiency of the system, the internal geometry was modified according to the split-and-recombine (SAR) model [41]. The idea of creating a long circumvolved path led to an increase in the surface-to-volume ratio, so that the surface area that can be running through by the phases can be extended without the need to increase the dimension [42]. The simplest MF devices are differentiated into T-shaped or Y-shaped depending on whether the angle of junction between the two fluids is 90° or less, respectively [38]. In our case, both 45° and 90° angles with square and round microchannels were structured to carry out a comparative study and assess whether the angle at which the two fluids meet influenced the result (Figure 3a,b,e,f). Thus, to implement chaotic advection, two more T-shaped square devices were designed with a shorter micromixing path, and the main channel was adorned with wedges and modified herringbone structure inside (Figure 3c,d) [43]. The most crucial characteristic of these MF devices relies on the fact that there is no evidence in the literature of DLP 3DP chips with the same complex and circumvolved internal path geometry.

All the properties of the DLP 3DP MFs’ devices can be found in Table 1.

### 3.2. Manufacturing of 3D DLP-Printed Devices

DLP 3DP is an extremely advanced technology that provides high printing precision while ensuring low costs and fast production times. It appears to be one of the most promising technologies to be applied to MF device production. Here, PlasCLEAR was used as photocurable resin due to its excellent visual clarity and transparency. This aspect is essential for MF devices to evaluate the exact microchannels’ dimension and the complexity of the internal geometry, as well as the presence of obstructions that might clog the microchannels [44].

The designs have been created using open-source online CAD software (TinkerCAD, USA). Once realized, they have been converted into an .STL format that is able to be supported by the slicing software associated with the DLP printer. Each device was designed with a rectangular shape flat at the bottom, which avoided the addition of supports during printing. This approach has made it possible to reduce manufacturing time (saving energy) and to limit the amount of resin used for each print, as the supporters cannot be reused once removed, thus demonstrating the superior sustainability of this production technique [20]. 

Again, *in-house* production and the availability of the 3D technique to independently print the various geometries made it possible to conduct a parameterized study of the various devices. The devices were labelled “diamond shaped” due to their macroscopic properties, as the transparency of the resin used and the intricate angled geometry of the microchannels create an optical resemblance to the articulated architecture of the diamond/crystal. Each device has a rectangular shape (28.00 × 50.00 × 2.70 mm) of which it is not possible to exceed the dimensional limits, due to the limit of the slicing software’s grid and the above-mentioned guidelines [40].

**Figure 4:**
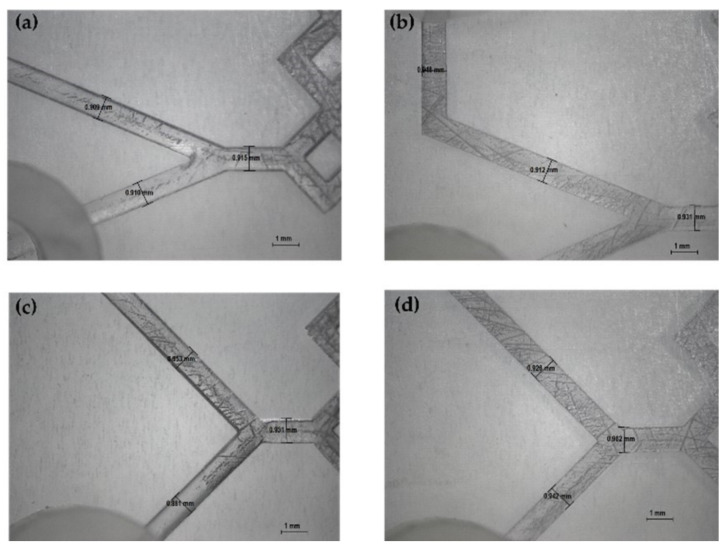
Optical microscope images of DLP 3DP devices (8×). (**a**) Inlet’s image of device 2. (**b**) Inlet’s image of device 6. (**c**) Inlet’s image of device 1. (**d**) Inlet’s image of device 5.

**Figure 5:**
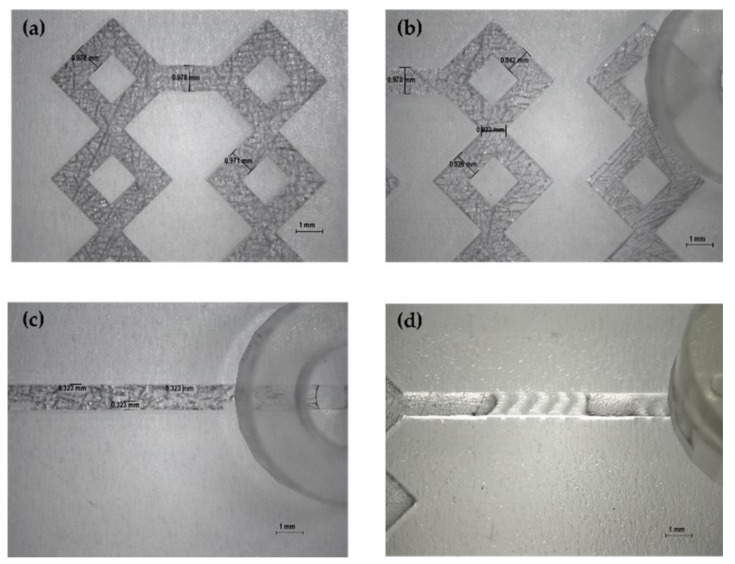
Optical microscope images of DLP 3DP devices (8×). (**a**,**b**) Image of the microchannels of devices. (**c**) Image of wedges (device 3). (**d**) Image of the modified herringbone structure (device 4).

The produced devices were subjected to various TFRs to test their resistance to increasing pressure. They are able to tolerate the TFR at 10 mL min^−1^ (the maximum in our system). This result represents an important proof of concept of the validity of the used method for the *in-house* production of MF devices. Representative device optical microscope images are shown in Figure 4 and Figure 5.

### 3.3. LPs’ Optimization and Production

Several studies were carried out to find the optimised operating conditions to achieve L-LPs. Empty nano-LPs were used as control to compare the various devices’ geometries and to set the best parameters regarding FRRs and TRFs. The first experiments involved the use of an organic solution consisting of DPPC/Chol (2:1) in ethanol at a final concentration of 1 mg mL^−1^. As widely reported in the literature, the presence of Chol in a 2:1 ratio (70–30%) to lipids asserts the optimum concentration ratio for stable and consistent vesicular systems [33]. Starting from a previous study in which several FRRs (aqueous phase: organic phase) were used to try to produce LPs via MFs [32], it was chosen to test all devices by applying different FRRs. In this case, in addition to defined best FRR (5:1) for LPs [32], 5:0.5 and 5:3 FRRs were used to try to optimize the operating conditions in order to achieve nanosized monodisperse LPs. The obtained data are shown in Table 2.

Tests conducted in obtaining empty nano-LPs as a control were used as proof of concept of the validity of the devices applied to MF *in-flow* nanoformulations’ production. Once the various devices in the production of nanoLPs composed of DPPC alone had been tested, the complexity of the LPs was increased by using a PEGylated phospholipid to decorate the nanosystems, improving them with long-circulating stealth properties [45]. Based on the optimization results from a previous study [32], the same operating conditions were used for the lipid solution. An organic solution of DPPC/16:0 PEG2000 PE/Chol (2:2:1) at a final concentration of 1 mg mL^−1^ was used, while the aqueous phase was PBS (pH 7.4).

**Figure 6:**
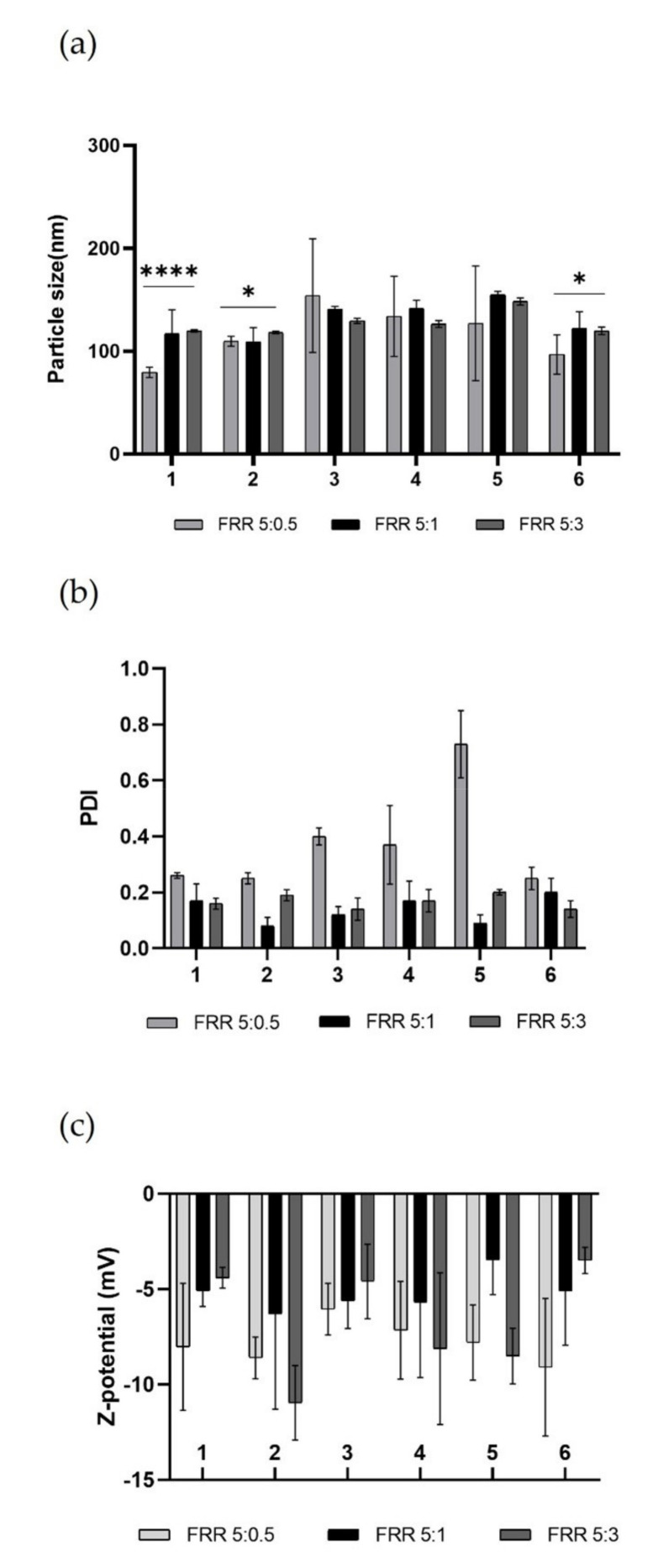
Representative graphs of empty LPs produced using DPPC/16:0 PEG2000 PE/Chol (2:2:1) in ethanol at total concentration 1mg mL^−1^. (**a**) Comparison of particle size (nm) of obtained LPs using different devices at several FRRs. To compare size, one-way ANOVA analysis was performed, adhering to a *p* value ≤ 0.05. In accordance with the results, comparing all devices for each FRRs, it was found that there was a significant difference (reported on the graph). Comparing each device for all FRRs, it was found that there was a significant statistical difference (****, *p* value < 0.0001) for device 1, while for device 2 and device 6 the statistical difference was modest (*, *p* value < 0.0332). (**b**) Comparison of PDI of obtained LPs using different devices at several FRRs. (**c**) Comparison of Z-potential (mV) of obtained LPs using different devices at several FRRs. Results represent mean ± SD, n = 3 independent batches.

The produced empty LPs were used to evaluate the quality of the various devices in the production of more complex nanosystems, to discriminate which were the best-operating conditions to be used subsequently for the encapsulation of the biologically active molecule. The characterization data of the achieved LPs are shown in Figure 6.

The resulting data revealed the high performance of all printed devices. All batches of LPs have optimal dimensions for drug administration [46]. By comparing the data from the literature, it was shown that LPs made using these DLP 3DP devices have lower size and PDI than those made using traditional methods [47,48].

The nature of the lipid composition massively influences the size of the produced LPs, and it has been demonstrated that vesicular systems produced using a single-lipid mixture are on average smaller than those produced using a double-lipid mixture, due to the more complete packing that occurs in the single-lipid mixture [39]. Despite this, no considerable differences were noted between the LPs obtained using (DPPC/Chol) single-lipid mixture and (DPPC/16:0 PEG2000 PE/Chol) double-lipid mixture in terms of size and PDI at fixed FRR and TFR, thus demonstrating the proficiency of the *in-house* micromixing geometry. It was also clarified that the diverse angle in which the two phases met did not affect the quality of achieved LPs, eliminating this parameter as a variable to consider. During nanoformulation production, it is crucial to consider the key role of TFR and FRR, which are the microfluidic parameters that massively influence the quality of LPs. Generally, an increase in TFR and FRR leads to an improvement in the quality of the obtained product [49]. Examining the results from the various FRRs used (Figure 6a), it was noted that raising the TFR does not guarantee an increase in the quality of the LPs, as the mixing between the phases is hugely due to the internal geometry that promotes chaotic aversion. The formulation with the narrower size is FRR 5:0.5. During the formation process of LPs, water-insoluble lipids are solubilized in an organic solvent, in this case ethanol, and the formation process takes place upon encountering the aqueous phase. The process of self-assembly of the lipid structure occurs due to the increased polarity of the solvent when the organic phase meets and mixes with the aqueous phase [50]. Thermodynamically semi-stable self-assembled structures are thus formed and are called bilayered phospholipid fragments (BPFs) [51]. BPFs turn into closed vesicular structures, increasing the surface energy of the system. If the ethanol concentration around the BPFs reduces, the BPFs grow to a thermodynamically stable size and decrease the energy of the system. If ethanol dilution is fast, as in the case of higher FRR, the size of the LPs is smaller but the system is not stable [52], resulting in high PDI as in the case of FRR 5:0.5 (Figure 6b). The composition of the liposomal membrane structure has been altered due to the FRR change, which is reflected by the change in PDI. Resulting LPs from FRR 5:1 and FRR 5:3 showed a high-quality size and performed better in terms of PDI. As expected, the Z-potential of the empty LPs (Figure 6c) was stable and negative due to the nature of the lipids used [32]. In this case, this characterisation parameter was not predictive about the quality of the formulation. Although the FRR 5:0.5 formulation provided a more stable negative potential on average, this was due to the smaller size and PDI of the LPs, which exhibited a higher surface area-to-volume ratio and thus a larger negative surface area. However, obtained LPs from FRR 5:1 and FRR 5:3 showed very similar Z-potential values.

On the basis of the previous data, *in-house* device 1 and device 4 were chosen for subsequent studies of lysozyme encapsulation within LPs (L-LPs). Devices were tested at both FRR 5:1 and FRR 5:3. Several concentrations of lysozyme were employed in the aqueous phase, namely 0.3, 0.5, and 0.7 mg mL^−1^.

**Figure 7:**
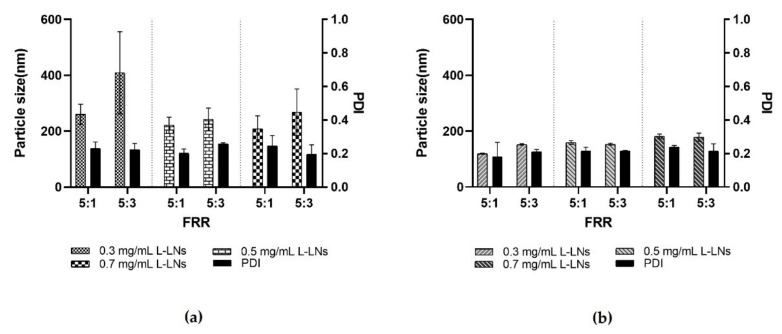
Resulting data in the production of L-LPs using several concentrations of lysozyme (0.3, 0.5, and 0.7 mg mL^−1^) at both FRR 5:1 and 5:3. (**a**) Achieved data using device 1. (**b**) Achieved data using device 4. Results represent mean ± SD, n = 3 independent batches.

As shown in Figure 7, both devices 1 and 4, performed extremely well. In the case of device 1, comparing the two different FRRs, a significant difference related to the size of L-LPs emerged. Device 1 has a geometry based on a massive micromixer that enables the LPs’ rapid self-assembly. The difference in size depends on the FRR used, as in the case of the FRR 5:3, the reduced amount of aqueous phase was not sufficient to adequately disperse the produced L-LPs, resulting in an aggregation process. In the other case, the FRR 5:1 allowed the nanosystems to stabilize due to the major presence of aqueous phase [53].

Device 4 was highly accomplished at both FRRs. The presence of the main microchannel with the modified herringbone structure inside after the micromixing step made it possible to steady the produced L-LPs, even in the case of a FRR 5:3 under the conditions of a smaller amount of aqueous continuous phase. The massive chaotic advection generated within device 4 causes the LPs to quickly self-assemble as the meeting of the two phases occurs and the polarity of the medium increases. Thus, LPs of minimal size are formed, whose monodispersity increases as the mixing time decreases [54].

L-LPs obtained using device 1 and device 4 have undergone subsequent stability studies. FRR 5:1 was chosen as most performing over FRR 5:3 due to the greater stabilization provided by the higher dispersion.

### 3.4. Stability Studies

**Figure 8:**
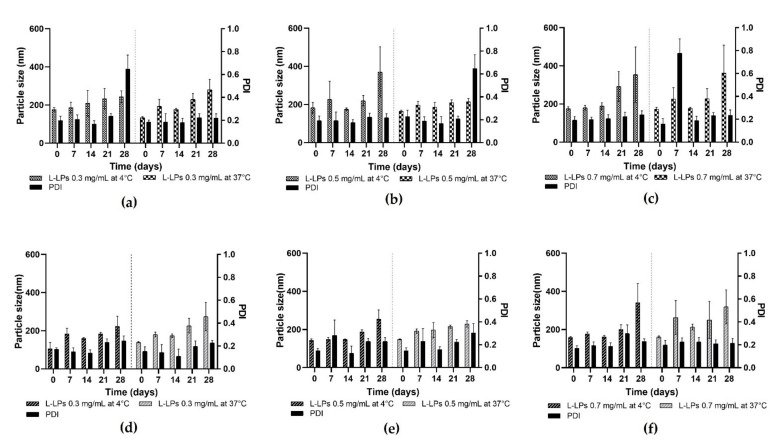
Stability profiles of L-LPs produced by device 1 and device 4. (**a**–**c**) Stability studies of 0.3, 0.5, and 0.7 mg mL^−1^ L-LPs at 4 and 37 °C using device 1; (**d**–**f**) 0.3, 0.5, and 0.7 mg mL^−1^ L-LPs at 4 and 37 °C using device 4. Results represent mean ± SD, n = 3 independent batches.

Variability in size plays a key role in the study of LPs’ physical stability assessment. L-LPs were produced using a mix of phospholipids characterised by saturated aliphatic chains. This chemical property leads to an increase in the stability of the nanosystems due to the Van der Waals interactions that are arranged in systematic manner along the bilayer structure and make it more rigid [40]. In this case, the fluidity of the membrane is guaranteed by the presence of Chol, which ensures the right balance between rigidity/flexibility [55,56]. Formulation stability studies were conducted using the lysozyme concentrations (0.3, 0.5, and 0.7 mg mL^−1^) tested for the production of L-LPs (FRRs 5:1). L-LPs produced using devices 1 and 4 were tested. Stability was assessed at fixed time points (day 0, 7, 14, 21, 28) at 4 and 37 °C. Stability at 4 °C is an important parameter to validate the physical storage stability of the formulations, while studies conducted at 37 °C were suitable to mimic the physical instabilities that might be generated at increasing temperature. The results are shown in Figure 8.

At 4 °C, an increase in the size of the L-LPs produced with device 1 is noted (Figure 8a–c), which starts early and maintains an almost constant trend until day 28. Concerning L-LPs produced with device 4, on the other hand, less of a growth trend is shown, which becomes more pronounced in the last week of analysis (Figure 8d–f). Stability at 37 °C was assessed for the formulations produced with both devices to analyse any changes that may affect phospholipids at higher temperatures (Figure 8). Although the growth trend of the LPs is more pronounced than at storage temperature, the L-LPs maintain a size available to drug administration [46]. Although physical stability tests were successful for both device formulations, device 4 proved to be the better performer in terms of produced L-LPs.

### 3.5. EE% and In Vitro Release Study

From previously illustrated data (Figure 7), it was found that device 4 was more reliable for the production of monodisperse nanosized L-LPs. For this reason, tested L-LPs formulations to evaluate the encapsulation efficacy were produced using device 4.

**Figure 9:**
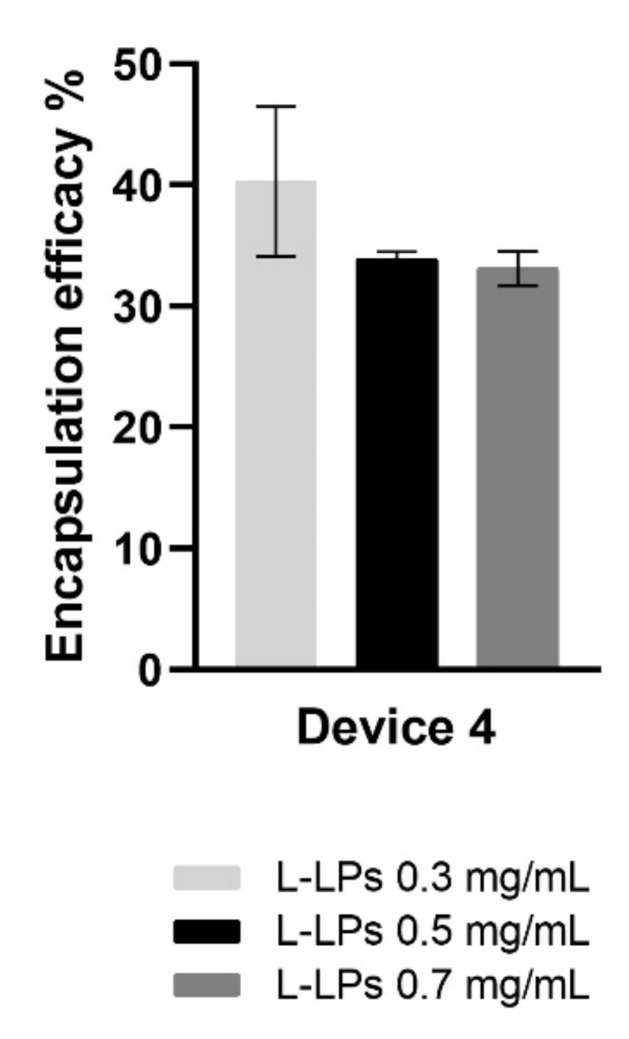
EE (%) of L-LPs encapsulating several lysozyme concentrations (0.3, 0.5, and 0.7 mg mL^−1^) produced using device 4. Results represent mean ± SD, n = 3 independent batches.

It was clearly shown that a higher EE% at 40.89 ± 6.19% was achieved for the L-LPs 0.3 mg mL^−1^ formulation than for the L-LPs 0.5 mg mL^−1^ and L-LPs 0.7 mg mL^−1^ formulations, which achieved both circa 30% (Figure 9).

Assessments from the stability studies showed that the L-LPs 0.3 mg mL^−1^ formulation was less stable than the others, both in terms of size and PDI. Taking this evidence into account, it was decided to conduct the in vitro release study by investigating the profile generated by the L-LPs 0.5 mg mL^−1^ formulation, which was found to be better performing.

**Figure 10:**
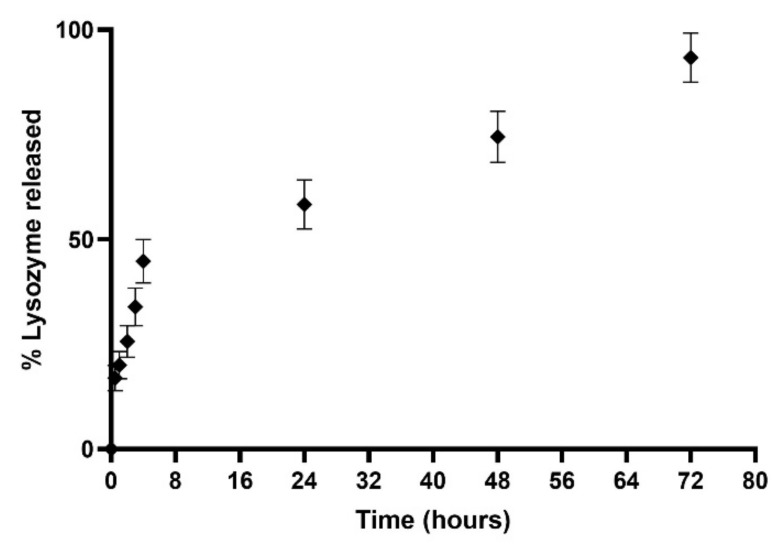
Cumulative (%) release study profile of device 4-produced L-LPs 0.5 mgmL^−1^ conducted in PBS (pH 7.4) at 37 °C. Results represent mean ± SD, n = 3 independent batches.

The release study was conducted under dynamic dialysis for three days at 37 °C in PBS (pH 7.4). Resulting data are displayed in Figure 10. The obtained release profile exhibited that the L-LPs 0.5 mg mL^−1^ formulation allowed for 93.36 ± 5.85% release of the total payload until 72 h of incubation. The profile showed an initial rapid release up to 44.82 ± 5.16% in the first 4 h of incubation, and then, the curve turned into a zero-order kinetics release (R^2^ = 0.9985) [57], which performed a controlled release over time. This kind of combined-kinetics release could be explained taking into account the placement of drugs within LPs, considering the fast release of lysozyme placed next to the lipid bilayer instead of the slow and constant path of drug located within the LPs aqueous core [58]. In comparison with a previous study [32], there was a slightly higher release explained by considering the lower molecular weight of the considered drug (≅14 kDa) [59]. In particular, for LPs, it is crucial to consider the type of aliphatic chain constituting the phospholipids and the transition temperature associated with them; thus, it may influence the release profile at physiological temperatures [60]. In addition, a major aliphatic chain was shown to result in the formation of additional hydrophobic and Van der Waals interactions that stabilize the structure and protect the drug from faster release over time [61].

This point opens the way for possible additional in-depth studies to evaluate whether the use of different phospholipids could cause variations in the profile shown here, leading to formulation customization depending on the type of target and desired release profile.

## 4. Conclusions and Future Perspectives

It is critical to investigate new ways for the continuous and repeatable manufacture of unique formulations appropriate for medical therapy. Moreover, it is suitable to explore the delivery of biologically active molecules, which represents a significant advancement in the field of nanomedicine. Despite the fact that there have been several research studies on this subject, the evidence in the literature remains inconclusive. Here, a technical strategy was developed that involves the combination of MF and 3DP to achieve this goal. The result of the synergy between these two unique approaches is an important proof of concept for merging these two methodologies. Furthermore, the introduction of a novel and high-performance printing method, such as DLP, to the customized fabrication of MF devices offers a breakthrough with enormous promise. One of the most significant is the ability to independently develop MF devices and receive it quickly and cheaply. Here, using *in-house* designed and printed diamond-shaped devices, the feasibility of producing high-performing stealth LPs carrying lysozyme as a model enzyme was highlighted. Achieved LPs, both empty and carrying the model drug, were extremely well performing in terms of size and PDI, with great reproducibility of the method. Stability studies showed that the formulations produced with devices 1 and 4 remained stable over time and suitable for administration. The defined better device (device 4) allowed L-LPs with good EE% to be obtained. In vitro release study performed with L-LPs produced using the better device 4 illustrated an almost complete release of the total cargo within 72 h of incubation. 

Although this study is still exploratory, taking advantage of the association between 3DP and MF, the promise of this synergy is extremely high, paving the way for the usability of innovative formulations that represent the future of nanomedicine.

## Figures and Tables

**Table 1:** Summary of the DPL 3DP MF device properties.

Device	Angle (°)	Channel Type	Geometry	Dimensions (mm)	Internal Geometry (mm)
**1**	90	Round	Diamond	1.0 × 1.0 × 2.30	-
**2**	45	Round	Diamond	1.0 × 1.0 × 2.30	-
**3**	90	Square	Diamond + wedges (internal)	1.0 × 1.0 × 2.30	0.8 × 0.8 × 0.8
**4**	90	Square	Diamond + herringbone (internal)	1.0 × 1.0 × 2.30	0.8 × 1.94 × 1.22
**5**	90	Square	Diamond	1.0 × 1.0 × 2.30	-
**6**	45	Square	Diamond	1.0 × 1.0 × 2.30	-

**Table 2:** Summary overview of obtained data by testing all devices with several FRRs. One-way ANOVA analysis was performed adhering to a *p* value ≤ 0.05. In accordance with the results, comparing all devices for each FRRs, it was found that there was a significant difference (*p* value < 0.0001). Comparing each device for all FRRs, it was found that there was a statistical difference (*p* value < 0.0001) for all devices. Results represent mean ± SD, n = 3 independent batches.

Device	FRRs (PBS: Organic Phase)					
	5:0.5		5:1		5:3	
	Particle Size (nm)	PDI	Particle Size (nm)	PDI	Particle Size (nm)	PDI
**1**	111.55 ± 4.56	0.19 ± 0.03	181.42 ± 11.53	0.17 ± 0.04	148.21 ± 4.77	0.08 ± 0.03
**2**	123.39 ± 2.21	0.19 ± 0.02	180.37 ± 11.35	0.22 ± 0.01	141.15 ± 1.72	0.09 ± 0.04
**3**	136.71 ± 12.07	0.21 ± 0.02	204.44 ± 36.11	0.16 ± 0.04	171.25 ± 2.58	0.08 ± 0.04
**4**	125.62 ± 4.33	0.14 ± 0.03	169.08 ± 8.63	0.19 ± 0.04	140.97 ± 2.46	0.12 ± 0.03
**5**	194.71 ± 38.86	0.20 ± 0.04	302.55 ± 20.72	0.23 ± 0.01	164.50 ± 9.83	0.16 ± 0.04
**6**	134.38 ± 5.12	0.22 ± 0.01	147.86 ± 2.58	0.17 ± 0.02	152.84 ± 2.00	0.09 ± 0.02

## Data Availability

Data available on request due to restrictions, e.g., privacy or ethical.
